# Deciphering the molecular nexus between Omicron infection and acute kidney injury: a bioinformatics approach

**DOI:** 10.3389/fmolb.2024.1340611

**Published:** 2024-07-04

**Authors:** Li Wang, Anning Chen, Lantian Zhang, Junwei Zhang, Shuqi Wei, Yangxiao Chen, Mingliang Hu, Yihao Mo, Sha Li, Min Zeng, Huafeng Li, Caixing Liang, Yi Ren, Liting Xu, Wenhua Liang, Xuejiao Zhu, Xiaokai Wang, Donglin Sun

**Affiliations:** ^1^ Nephrology Department, Southern Medical University Affiliated Longhua People’s Hospital, Shenzhen, China; ^2^ Affiliated Cancer Hospital and Institute of Guangzhou Medical University, Guangzhou, China; ^3^ Department of Anesthesiology, The Second Affiliated Hospital of Soochow University, Suzhou, Jiangsu, China; ^4^ Xuzhou First People’s Hospital, Xuzhou, Jiangsu, China; ^5^ Department of Urology, Shenzhen Hospital, Southern Medical University, Shenzhen, China

**Keywords:** COVID-19, Omicron infection, acute kidney injury, disease biomarker, hub gene

## Abstract

**Background:**

The ongoing global health crisis of COVID-19, and particularly the challenges posed by recurrent infections of the Omicron variant, have significantly strained healthcare systems worldwide. There is a growing body of evidence indicating an increased susceptibility to Omicron infection in patients suffering from Acute Kidney Injury (AKI). However, the intricate molecular interplay between AKI and Omicron variant of COVID-19 remains largely enigmatic.

**Methods:**

This study employed a comprehensive analysis of human RNA sequencing (RNA-seq) and microarray datasets to identify differentially expressed genes (DEGs) associated with Omicron infection in the context of AKI. We engaged in functional enrichment assessments, an examination of Protein-Protein Interaction (PPI) networks, and advanced network analysis to elucidate the cellular signaling pathways involved, identify critical hub genes, and determine the relevant controlling transcription factors and microRNAs. Additionally, we explored protein-drug interactions to highlight potential pharmacological interventions.

**Results:**

Our investigation revealed significant DEGs and cellular signaling pathways implicated in both Omicron infection and AKI. We identified pivotal hub genes, including EIF2AK2, PLSCR1, GBP1, TNFSF10, C1QB, and BST2, and their associated regulatory transcription factors and microRNAs. Notably, in the murine AKI model, there was a marked reduction in EIF2AK2 expression, in contrast to significant elevations in PLSCR1, C1QB, and BST2. EIF2AK2 exhibited an inverse relationship with the primary AKI mediator, Kim-1, whereas PLSCR1 and C1QB demonstrated strong positive correlations with it. Moreover, we identified potential therapeutic agents such as Suloctidil, Apocarotenal, 3′-Azido-3′-deoxythymidine, among others. Our findings also highlighted a correlation between the identified hub genes and diseases like myocardial ischemia, schizophrenia, and liver cirrhosis. To further validate the credibility of our data, we employed an independent validation dataset to verify the hub genes. Notably, the expression patterns of PLSCR1, GBP1, BST2, and C1QB were consistent with our research findings, reaffirming the reliability of our results.

**Conclusion:**

Our bioinformatics analysis has provided initial insights into the shared genetic landscape between Omicron COVID-19 infections and AKI, identifying potential therapeutic targets and drugs. This preliminary investigation lays the foundation for further research, with the hope of contributing to the development of innovative treatment strategies for these complex medical conditions.

## 1 Introduction

Acute Kidney Injury (AKI) is a clinical condition characterized by a swift decrease in the rate of glomerular filtration, resulting in the build-up of metabolic byproducts (Levey and James, 2017). This condition correlates with elevated risks of mortality, cardiovascular events, and progression to chronic kidney disease (McCullough et al., 2016; Levey and James, 2017). AKI severity is classified based on urine output and creatinine level increases, with etiologies grouped into prerenal, intrinsic renal, and postrenal categories (McCullough et al., 2016; Peerapornratana et al., 2019). Precise diagnosis of the root cause is crucial for effective management.

The Omicron variant of SARS-CoV-2 was initially detected in Botswana and South Africa in November 2021, which led to its classification as a variant of concern by the World Health Organization (Fan et al., 2022). Ongoing research seeks to understand its transmissibility and potential disease severity. After arriving in the United States in late 2021, Omicron spread rapidly, giving rise to multiple subvariants capable of evading immune responses (Fan et al., 2022; Kandeel et al., 2022). Researchers continue to monitor these subvariants, as well as hundreds of other Omicron progeny globally.

AKI and Omicron infections present significant public health challenges worldwide (Yende and Parikh, 2021). In light of the Omicron pandemic and AKI prevalence, concerns have emerged about the potentially heightened vulnerability of AKI patients to primary and secondary Omicron infections and their associated disease severity. A potential association between COVID-19 infection and the onset of AKI has been investigated. Research suggests that nearly 28% of patients admitted to the hospital for COVID-19 receive an AKI diagnosis, and about 9% of these cases necessitate renal replacement therapy (Yende and Parikh, 2021; Yan et al., 2023). SARS-CoV-2 infection exerts both direct and indirect effects contributing to AKI development (Yende and Parikh, 2021). Hence, it is imperative to ascertain the impact of primary and secondary Omicron infections on individuals afflicted with AKI and discern potential therapeutic agents that may confer benefits to Omicron-positive AKI patients, thereby reducing the likelihood of hospitalization or mortality.

In our research, we discovered several crucial gene networks and cellular signaling pathways commonly associated with both primary and secondary Omicron infections as well as AKI. We also identified four hub genes that could act as innovative indicators for personalized treatment in patients with COVID-19 and AKI. Our investigation further spotlighted potential therapeutic drug candidates that may be effective in treating this specific patient population. Using a methodology rooted in systems biology and bioinformatics, we explored potential molecular mechanisms and recognized an array of biomarkers and potential treatments that could be advantageous for patients affected by the Omicron variant of COVID-19 and AKI.

## 2 Materials and methods

### 2.1 Gene expression data

To unearth the shared genetic links and relationships between Omicron-infected and AKI-affected individuals, we made use of RNA-seq and microarray analysis data from the Gene Expression Omnibus (GEO) database (Barrett et al., 2013). We leveraged GEO accession number GSE201530 (1st Omicron infection) (Lee et al., 2022a) and GSE205244 (1st and 2nd Omicron infection) (Lee et al., 2022b) as the datasets for Omicron infection, which included information on peripheral blood mononuclear cells (PBMCs) from Omicron-infected patients and healthy controls. The AKI dataset (GSE1563) (Flechner et al., 2004) consisted of kidney biopsy samples from individuals with AKI and healthy counterparts. The kidney disease (KD) validation dataset (GSE66494) included 53 patients with KD and eight healthy controls. The sequencing data were collected using the Affymetrix Human Genome U95 Version 2 Array. Table 1 provides an exhaustive summary of the information contained within these datasets.

**TABLE 1:** Overview of the datasets with their geo-features and quantitative measurements in this analysis.

Disease name	Geo accession	GEO platform	Total DEGs count	Upregulated DEGs count	Downregulated DEGs count
1st Omicron infection	GSE201530	GPL24676	676	581	95
1st and 2nd Omicron infection	GSE205244	GPL24676	800	318	482
Acute kidney injury	GSE1563	GPL8300	156	136	20

### 2.2 Detection of differentially expressed genes (DEGs)

A gene is deemed differentially expressed when there is a statistically significant fluctuation in its transcriptional level under varying experimental scenarios (Mahmud et al., 2021). The core goal of this examination was to pinpoint DEGs within the Omicron infection and AKI datasets. Utilizing the “limma” package from the R software, along with the Benjamini–Hochberg correction for false-discovery rate management, we identified DEGs from lengthy expression values. We implemented cut-off criteria (adjusted *p*-value or *p*-value <0.05 and absolute value of log fold-change ≥1.0) to distinguish significant DEGs. We employed *Jvenn*, a web-based Venn-diagram tool (Bardou et al., 2014), to identify common DEGs across the datasets.

### 2.3 Gene ontology (GO) and pathway enrichment analysis

The “clusterProfiler” package in R was utilized to identify potential functions and pathways associated with the DEGs. GO and KEGG pathway analyses were conducted, applying a standardized metric (*p*-value <0.05, *Q*-value <0.25) to prioritize the top functional items and pathways.

### 2.4 Protein-Protein Interaction (PPI) network examination

We identified PPIs using the STRING database (Szklarczyk et al., 2019) and processed and analyzed the resulting PPI network with Cytoscape. Proteins encoded by the DEGs common to both Omicron infection and AKI datasets were used to build the PPI network. We employed the Markov cluster method of the STRING database to pinpoint gene clusters. The PPI network of frequently occurring DEGs was established using a composite score greater than 0.4. The CytoHubba plug-in for Cytoscape was employed to evaluate and explore significant nodes within the PPI network modules and to predict hub genes.

### 2.5 Gene Regulatory Network (GRN) assessment

In our study, we determined DEG-microRNA (miRNA) interaction networks and DEG-transcription factor (TF) interaction networks using the NetworkAnalyst tool (Xia et al., 2015). We used the TarBase (Sethupathy et al., 2006) and miRTarBase (Hsu et al., 2011) databases to elucidate DEG-miRNA interaction networks. The TF-DEG interaction networks were analyzed using the JASPAR database (Khan et al., 2018). We used common DEGs for the Gene Regulatory Network (GRN) analysis to shed light on the transcriptional components and miRNAs that govern DEGs at the post-transcriptional stage.

### 2.6 Assessment of potential drugs

We utilized DSigDB (Yoo et al., 2015), an extensive database, to pinpoint pharmacological compounds associated with DEGs. Through the Enrichr web server and the DSigDB database, we identified drugs enriched for DEGs common to both COVID-19 and AKI datasets, using an adjusted *p*-value <0.05 as the threshold for significance.

### 2.7 Gene-disease association evaluation

DisGeNET (Pinero et al., 2021) operates as a data management platform that amalgamates and normalizes information related to genes and variations associated with diseases from diverse sources. We employed NetworkAnalyst and DisGeNET to study gene-disease associations, revealing diseases and chronic conditions linked to the common DEGs.

### 2.8 Mouse model

We sourced 8-week-old male C57BL/6 mice from Rise Mice Biotechnology Co., Ltd. (Zhaoqing, China), each weighing approximately 20–25 g. These mice underwent renal ischemia-reperfusion (IR) injury or a sham procedure. To conduct the IR-AKI model (Chen J. et al., 2023), we first anesthetized the mice with a 1.25% 2,2,2-tribromoethanol injection into the peritoneal cavity. Following this, a dorsal incision was made, and IR was initiated by clamping the renal pedicle of both kidneys for 30 min, after which the clamps were removed. For the sham procedure, we only exposed the kidneys without any clamping. All procedures involving animals were sanctioned by the Animal Experimentation Committee at Rise Mice Biotechnology Co., Ltd. (Project ID: Rise mice-202306020007) and were carried out in compliance with the guidelines set by the Guide for the Care and Use of Laboratory Animals.

### 2.9 Statistical analysis

All statistical evaluations were executed using R software and Bioconductor. A *p*-value below 0.05 was deemed to denote statistical significance.

## 3 Results

### 3.1 Detection of genetic links between AKI and COVID-19

**FIGURE 1:**
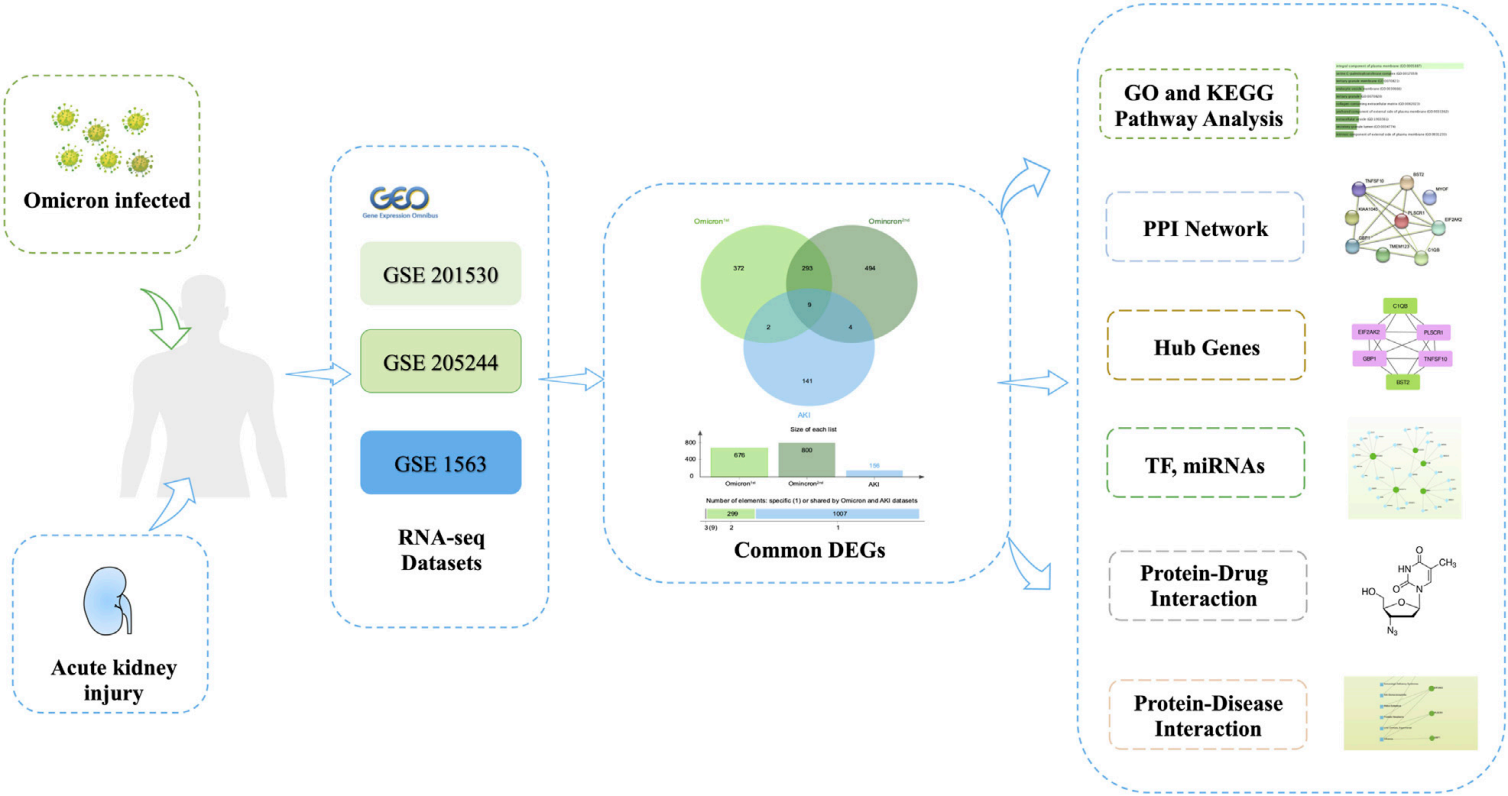
The comprehensive workflow employed in this study, providing an overview of the bioinformatic processes and data analysis steps conducted.

**FIGURE 2:**
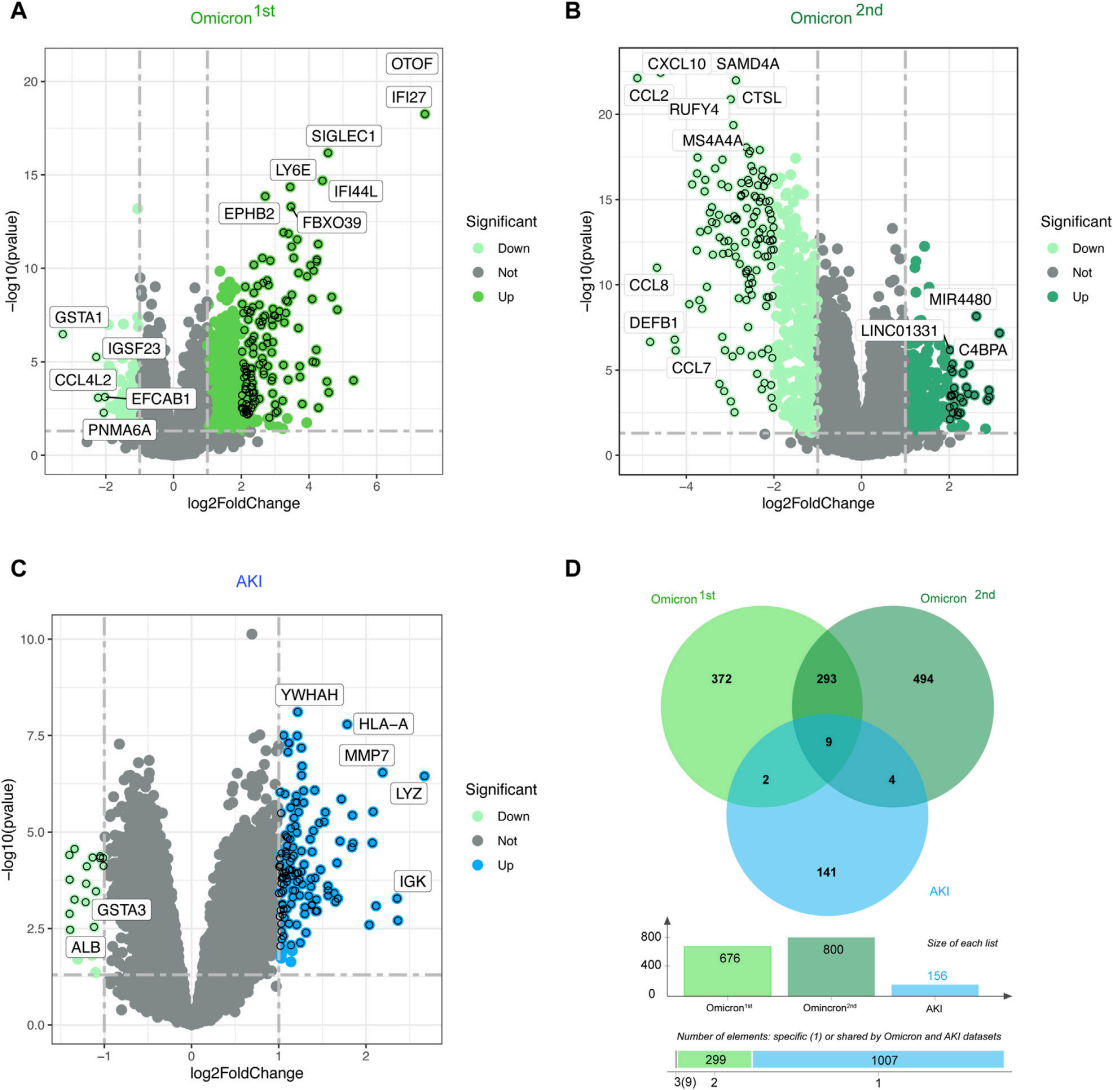
Volcano plots representing the gene expression patterns in primary and secondary Omicron infections **(A, B)** as well as AKI **(C)**. Additionally, the Venn diagram **(D)** showcases the shared differentially expressed genes (DEGs) among AKI, primary Omicron infection, and secondary Omicron infection.

Figure 1 depicts the fundamental steps employed in this investigation. In order to explore the shared genetic associations between primary and secondary Omicron infections and AKI, we meticulously examined human RNA-seq and microarray datasets obtained from GEO. Through this analysis, we identified the mutually regulated DEGs that play a role in initiating Omicron infection and AKI. Initially, we scrutinized the transcriptomic datasets of patients with primary Omicron infection and observed 676 DEGs when compared to healthy controls. Additionally, we identified 800 DEGs between primary and secondary Omicron infections. Similarly, our analysis of the AKI dataset revealed 156 DEGs (Table 1). To visually represent the DEGs for Omicron infections and AKI, volcano plots were generated and displayed in Figures 2A–C. Furthermore, by employing Jvenn, a reliable web service for Venn analysis, we identified nine shared DEGs among both primary and secondary Omicron infections and the AKI dataset (Figure 2D). A comprehensive list of all common DEGs can be found in Supplementary Table S1.

### 3.2 Functional enrichment analysis reveals significant cellular signaling pathways and GO terms

**FIGURE 3:**
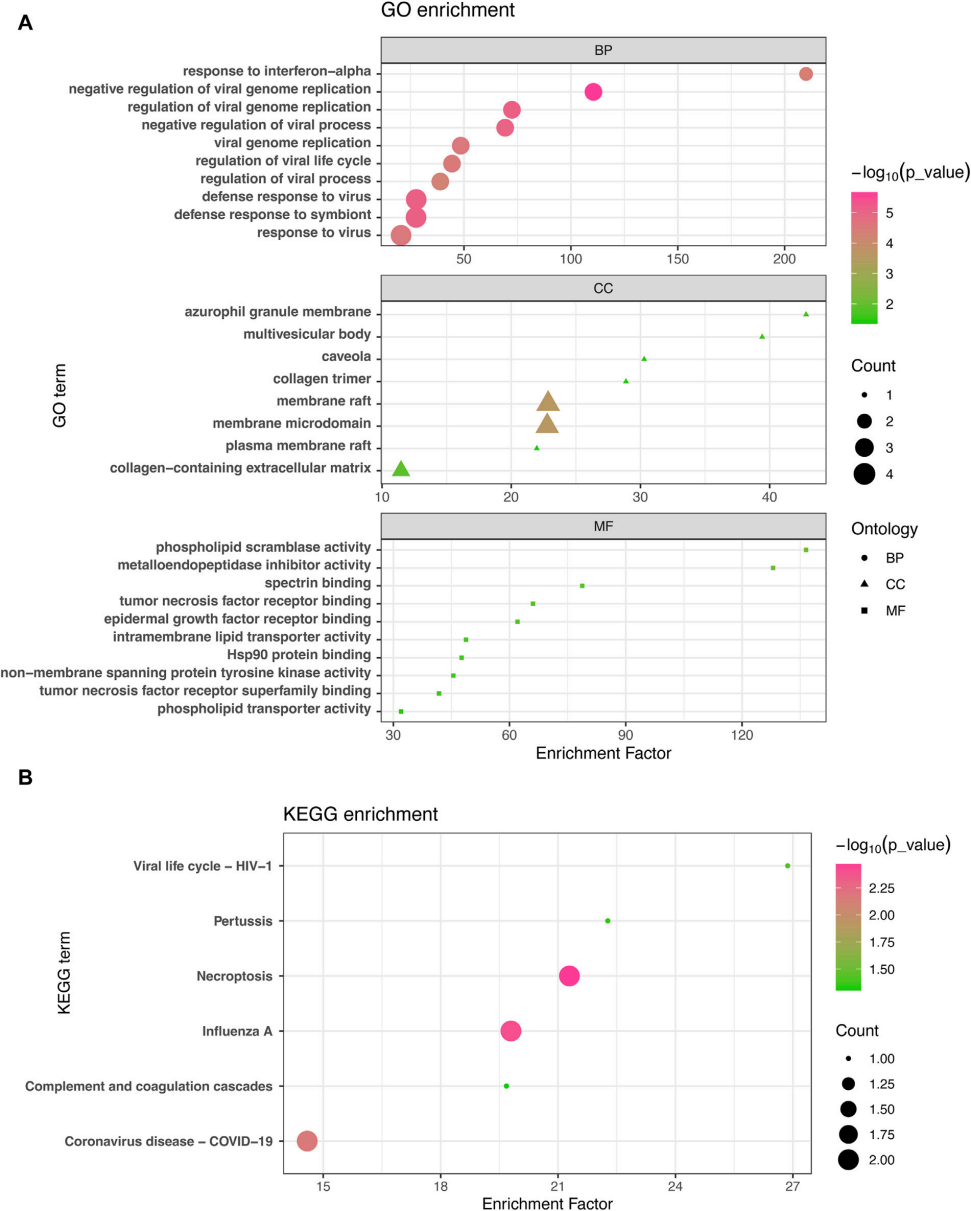
GO analysis **(A)** and KEGG analysis **(B)** were conducted to examine the shared differentially expressed genes among acute kidney injury, primary Omicron infection, and secondary Omicron infection.

The GO enrichment approach is a widely employed method to elucidate associations between genes and GO terms, while the KEGG enrichment approach enables the identification of gene-pathway associations. To accomplish this, we employed the “clusterProfiler” package to perform a functional enrichment test on the shared DEGs. The GO database served as an annotation source for the GO analysis, encompassing three categories: biological processes, cellular components, and molecular functions. Our findings demonstrated significant enrichment of DEGs in response to interferon-alpha in the biological process category, azurophil granule membrane in the cellular component category, and phospholipid scramblase activity in the molecular function category (Figure 3A). Furthermore, our KEGG results unveiled the top six pathways associated with the shared DEGs, including Viral life cycle-HIV-1, Pertussis, Necroptosis, Influenza A, Complement and coagulation cascades, and COVID-19 (Figure 3B).

### 3.3 PPI analysis discovers functional networks

**FIGURE 4:**
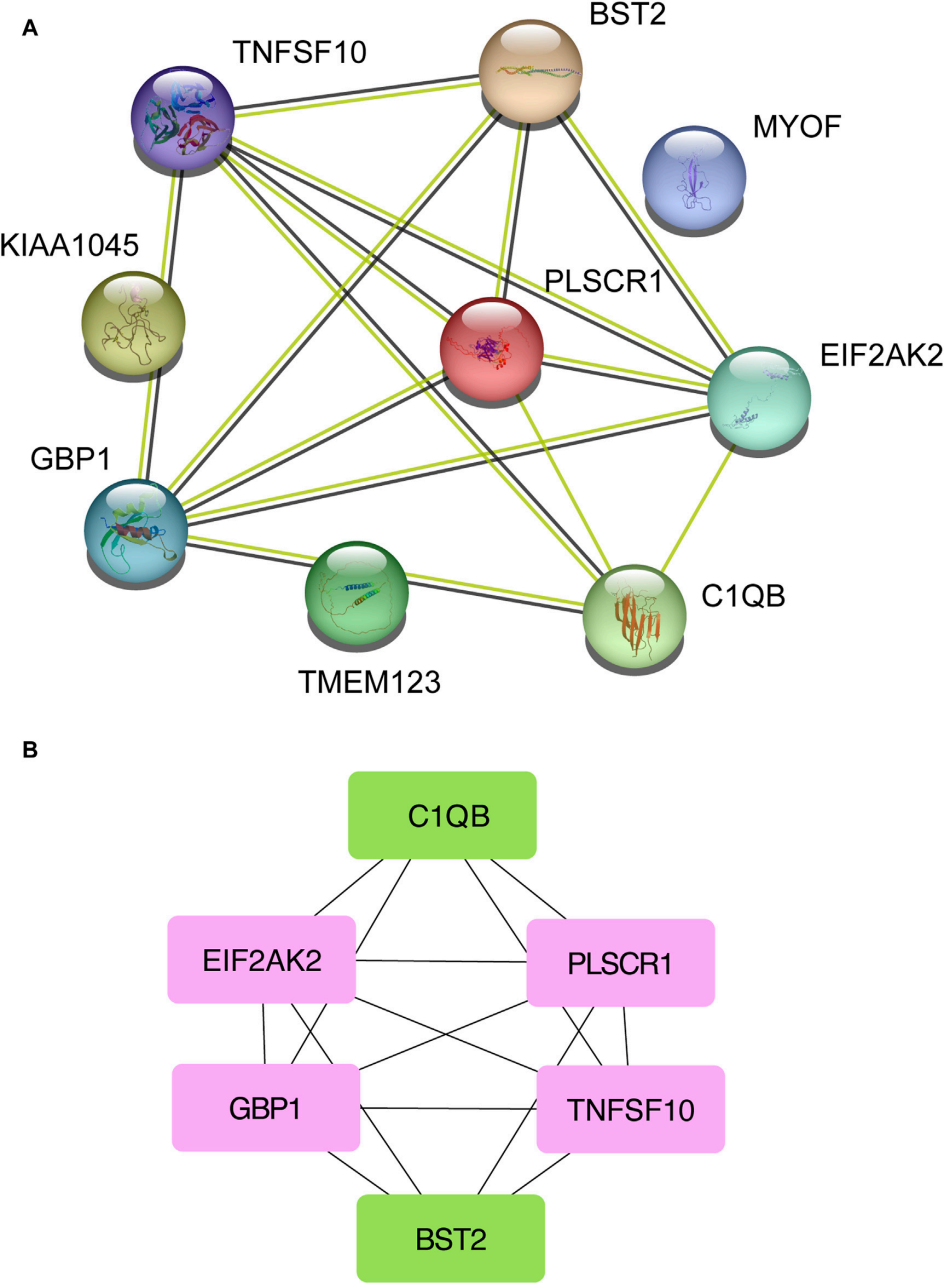
A Protein-Protein Interaction network **(A)** was constructed to analyze the hub genes **(B)** among the differentially expressed genes shared by acute kidney injury, primary Omicron infection, and secondary Omicron infection.

Next, the shared DEGs between the Omicron infection and AKI datasets were fed into the STRING database to investigate PPIs and identify joint DEG interactions and adhesion pathways. The PPI network of the shared DEGs between Omicron infection and AKI datasets is depicted in Figure 4A. The most highly interconnected nodes are designated as hub genes within the PPI network. Through the PPI network analysis performed using the Cytohubba plug-in for Cytoscape, we were able to identify the top six most influential DEGs, namely EIF2AK2, PLSCR1, GBP1, TNFSF10, C1QB, and BST2. These hub genes have the potential to serve as biomarkers leading to innovative treatment approaches for the diseases under study. We also utilized the Cytohubba plug-in to construct a submodule network to aptly depict the close relationships and the proximity of these genes (Figure 4B).

### 3.4 Validating the identified hub genes in a murine AKI model

**FIGURE 5:**
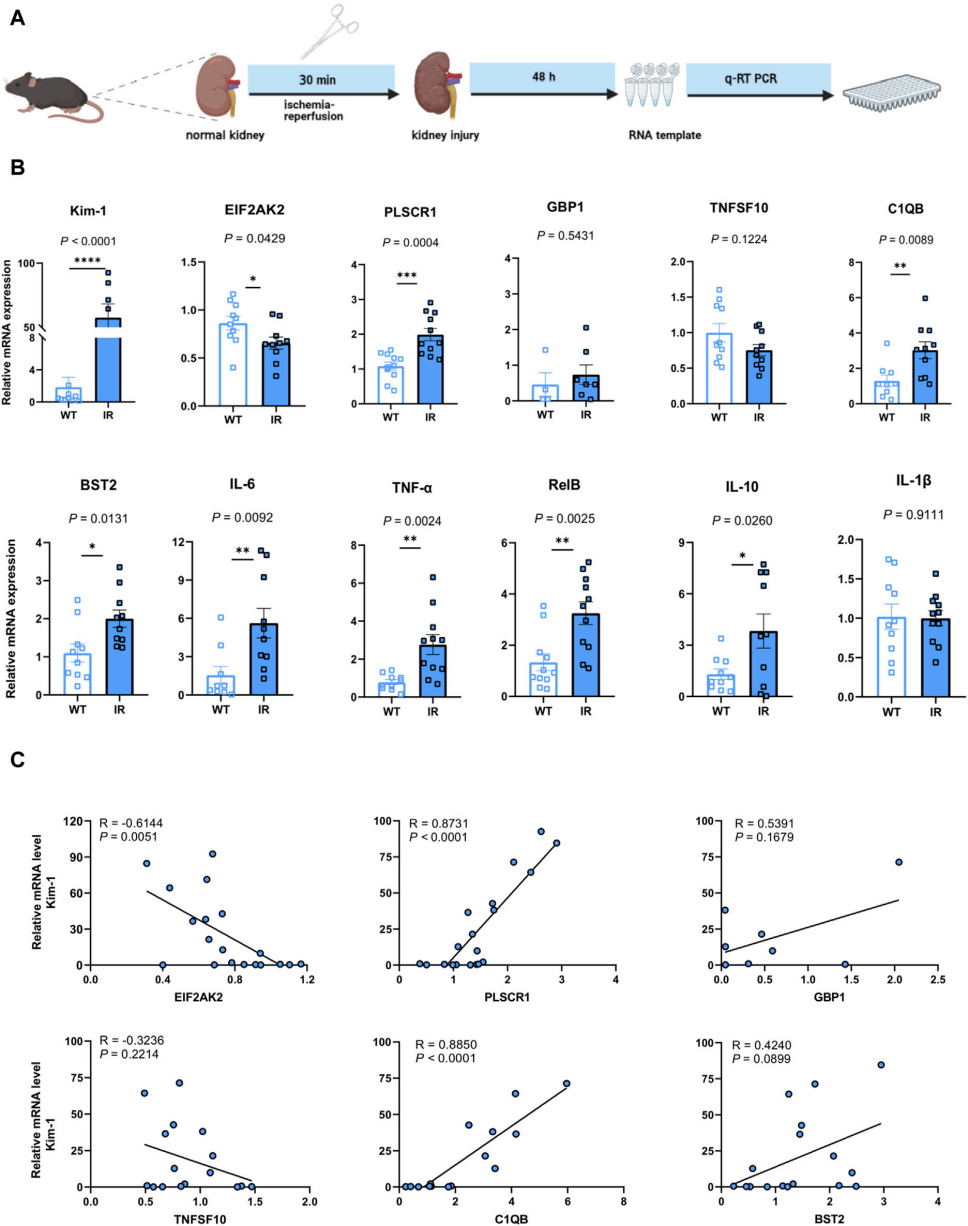
Confirmation of the pinpointed hub genes utilizing a mouse model of AKI. **(A)** A diagrammatic portrayal of the experimental method. **(B)** The quantities of cytokine mRNA found in homogenized kidney tissues (*n* = 4–11). Analysis was conducted using Student’s t-test. **(C)** A related scatterplot is provided, illustrating the associations between mRNA expression levels of the hub genes identified and Kim-1 in the kidney, determined via Spearman’s rank correlation (R).

To evaluate the potential application of the identified hub genes as predictive biomarkers for AKI, we employed murine models subjected to IR-induced AKI (Figure 5A). In comparison to the control group, the AKI mice showed substantial elevations in the levels of PLSCR1, BST2, C1QB, Kim-1, IFN-γ, TNF-α, RelB, and IL-10, whereas a decline was observed for EIF2AK2 (Figure 5B). Importantly, EIF2AK2 showed a negative relationship with the key AKI mediator, Kim-1 (Han et al., 2002; Ix and Shlipak, 2021), while PLSCR1, and C1QB manifested a positive correlation with the same (Figure 5C).

### 3.5 Networks GRN analysis uncovers DEG–miRNA- and TF–gene-interaction networks

**FIGURE 6:**
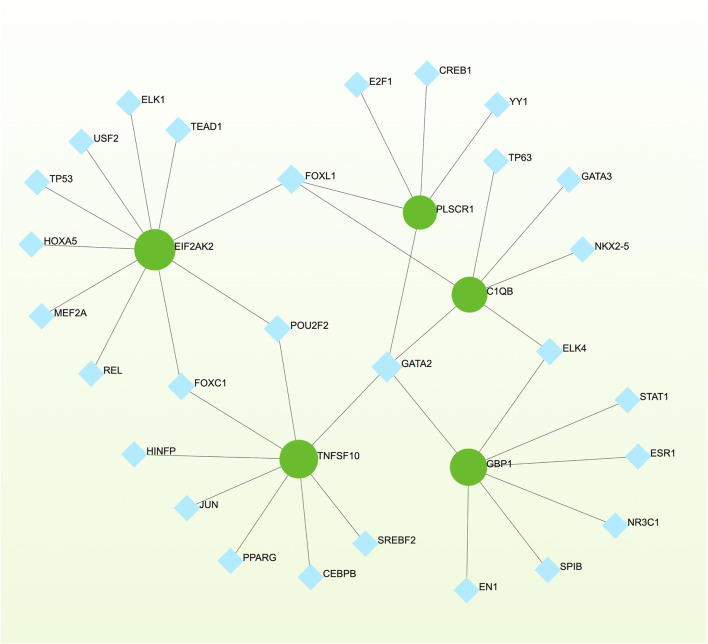
The regulatory interaction network, generated using Network Analyst, showcases the interconnected relationship between differentially expressed genes (DEGs) and transcription factors (TFs). In the network, TFs are represented by blue nodes, while the interaction between gene symbols and TFs is depicted by green nodes.

**FIGURE 7:**
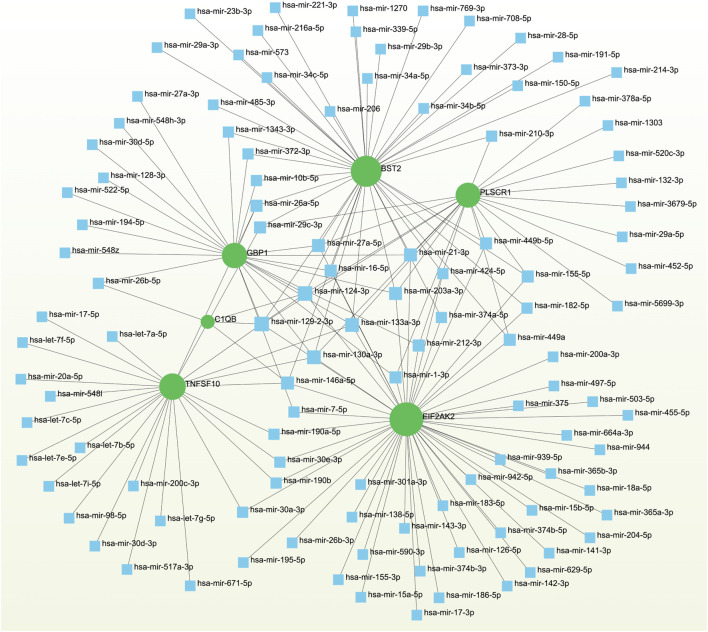
The regulatory interaction network highlights the interconnectedness between differentially expressed genes (DEGs) and miRNAs. In this network, TFs are denoted by blue square nodes, while the green circle node represents the gene symbols involved in interactions with miRNAs.

To pinpoint crucial transcriptional shifts and acquire a more comprehensive understanding of regulatory hub proteins, we employed network analysis to reveal regulatory TFs and miRNAs. The interactions between TF regulators and the detected hub genes are depicted in Figure 6, while the associations of miRNA regulators with the identified hub genes are illustrated in Figure 7. We found that 65 TFs and 119 post-transcriptional (miRNA) regulatory signals were anticipated to govern multiple detected hub genes, implying a significant interaction between them. The assembly and scrutiny of the regulatory target TF–gene and target miRNA–gene networks, as well as the topology table, are presented in Supplementary Tables S2, S3, respectively.

### 3.6 Detection of potential drugs

Evaluating protein-drug interactions helps to understand the structural characteristics beneficial for receptor sensitivity, which can be instrumental in the process of drug discovery (Mahmud et al., 2020). The hub genes identified based on interactions between Omicron infection and AKI were used in this examination. Using Enrichr, we identified ten potential therapeutic drugs based on transcriptional features from the DSigDB database, and the top 10 candidate compounds were drawn out according to their *p*-values. The top 10 enriched drugs in the DSigDB database (Suloctidil, Apocarotenal, 3′-Azido-3′-deoxythymidine, beta-carotene, Selenium, Vigabatrin, Prenylamine, Isoetarine, and Terfenadine) are detailed in Table 2.

**TABLE 2:** The recommended medications.

Name	*p-*value	Chemical formula	Structure
Suloctidil	9.67E-11	C_20_H_35_NOS	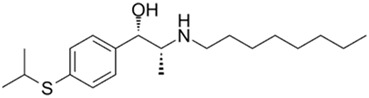
Apocarotenal	1.31E-06	C_30_H_40_O	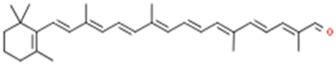
3′-Azido-3′-deoxythymidine	1.75E-06	C_10_H_13_N_5_O_4_	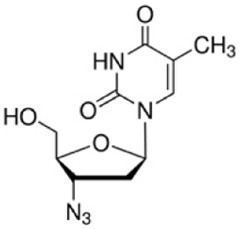
Beta-carotene	2.71E-06	C_40_H_56_	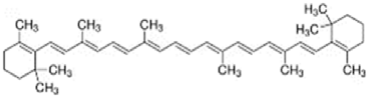
Selenium	1.61E-5	Se	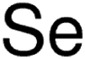
Vigabatrin	3.47E-5	C_6_H_11_NO_2_	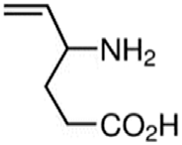
Prenylamine	5.24E-05	C_24_H_27_N	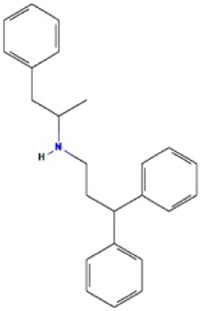
Isoetarine	9.87E-05	C_32_H_41_NO_2_	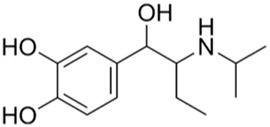
Terfenadine	1,31E-04	C_24_H_33_FO_6_	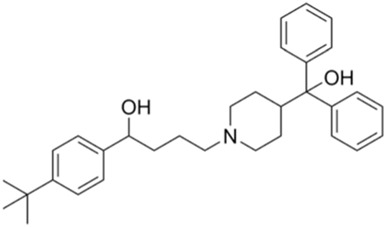

### 3.7 Detection of disease associations

**FIGURE 8:**
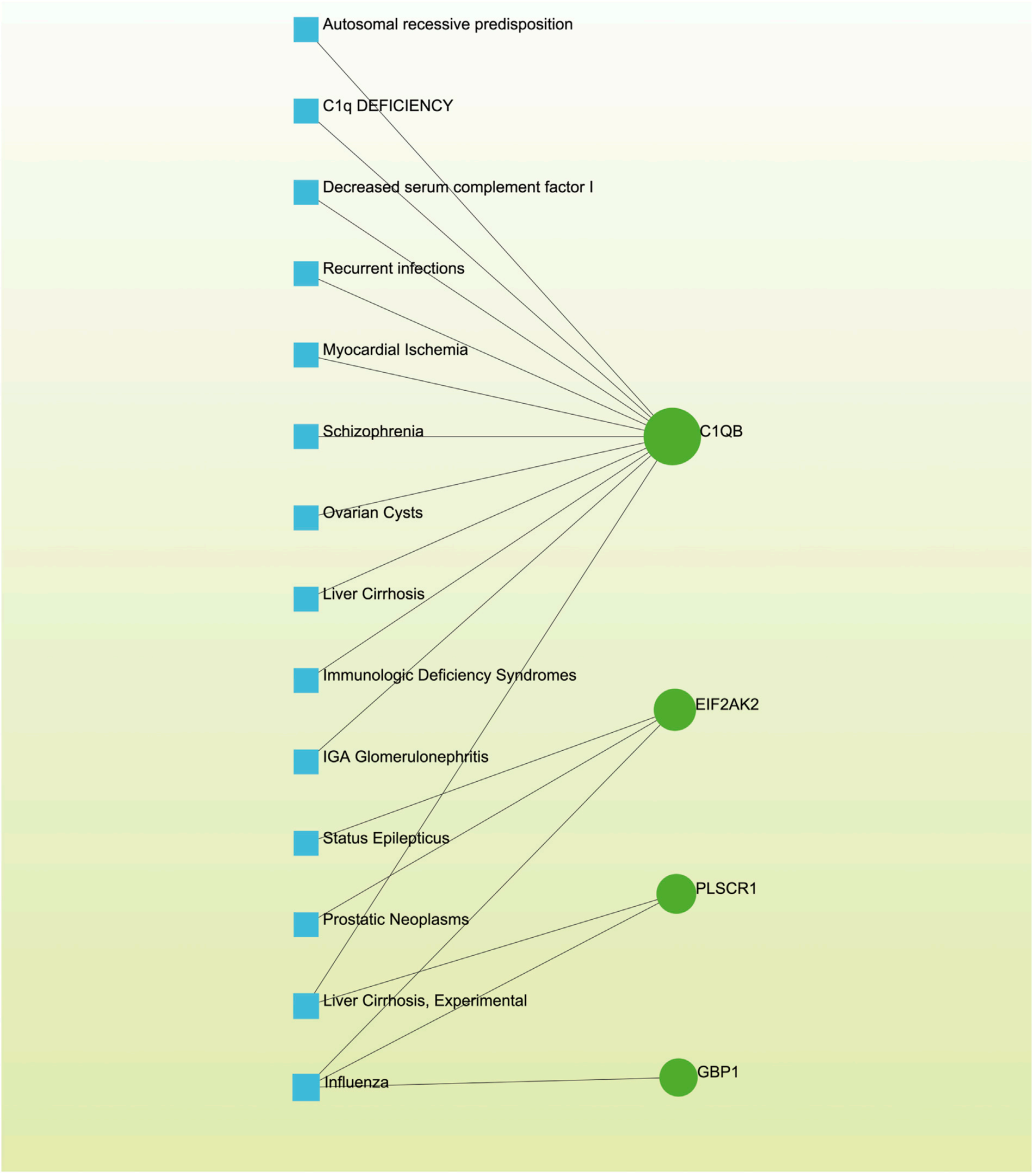
The gene-disease association network illustrates the connections between genes and diseases. In this network, diseases are represented by blue square nodes, while the gene symbols that interact with the respective diseases are denoted by green circle nodes.

Distinct diseases can exhibit connections or correlations under specific scenarios, such as when they possess one or more common genes (Nashiry et al., 2021). Decoding relationships between genes and diseases is an essential precursor to devising therapeutic interventions. Our investigation of gene-disease relationships via NetworkAnalyst divulged that Autosomal recessive predisposition, Myocardial Ischemia, Schizophrenia, Liver Cirrhosis, IgA Glomerulonephritis, Prostatic Neoplasms, and Influenza displayed the closest associations with the hub genes identified in this research (Figure 8).

### 3.8 Validation dataset

**FIGURE 9:**
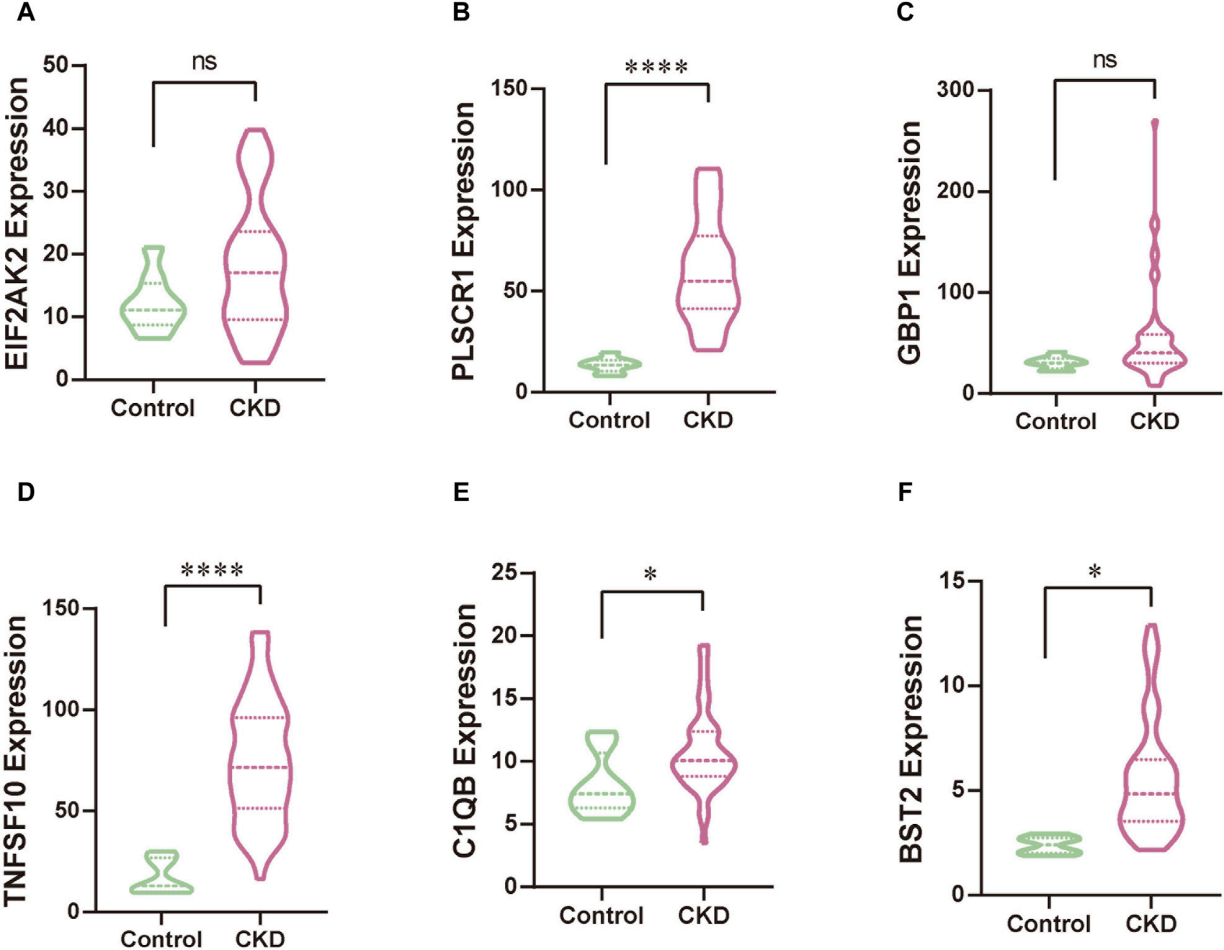
The validation of hub genes, specifically EIF2AK2 **(A)**, PLSCR1 **(B)**, GBP1 **(C)**, TNFSF10 **(D)**, C1QB **(E)**, and BST2 **(F)**, was conducted through rigorous statistical analysis utilizing Student’s t-test. This approach ensures the reliability and scientific rigor of our findings.

We leveraged a publicly available dataset to further validate the expression profiles of our focal hub genes in patients with KD. Through comparative analysis, we observed elevated expression levels of EIF2AK2, PLSCR1, GBP1, TNFSF10, C1QB, and BST2 in patients with KD (Figure 9). Among these results, the expression patterns of PLSCR1, GBP1, BST2, and C1QB were consistent with our initial research trends. However, the trends for EIF2AK2 and TNFSF10 deviated from our initial findings, which could be attributed to the comparatively intricate regulatory networks governing these genes. In our next steps, we aim to further explore the specific mechanisms underlying these discrepancies.

## 4 Discussion

Renal involvement is a frequent occurrence in individuals affected by acute Omicron infection, wherein subclinical inflammation and injury may persist over an extended period, leading to acute lung injury and a progressive decline in kidney function, ultimately culminating in chronic kidney disease (Yende and Parikh, 2021; Yan et al., 2023). Given the ongoing global burden of repetitive SARS-CoV-2 infections, particularly the Omicron variant, concerns have emerged regarding the heightened risk of AKI severity and exacerbations in individuals already afflicted with AKI (Yende and Parikh, 2021; Fan et al., 2022). High-throughput sequencing datasets have emerged as invaluable resources for identifying biomarkers associated with various disorders (Al-Mustanjid et al., 2020; Piazza et al., 2020). In this context, the integration of bioinformatics and systems biology analytics with patient sequencing data holds promise for enhancing our comprehension of the impact of SARS-CoV-2 on AKI patients, unveiling novel therapeutic strategies, and exploring alternative management approaches. In the present study, network analyses were employed to investigate gene-expression patterns derived from patient datasets encompassing AKI and primary and secondary Omicron infections. This approach enabled the identification of potential biomarkers for Omicron infections and AKI, as well as candidate drugs.

We analyzed transcriptomics data from AKI and primary and secondary Omicron infections and found nine common DEGs. These DEGs have biological relevance, as they are involved in key pathways during AKI and Omicron infections. These pathways include “response to interferon-alpha,” “azurophil granule membrane,” and “phospholipid scramblase activity.” These pathways may indicate the possible roles of these DEGs in the pathogenesis and progression of AKI and Omicron infections.

The “response to interferon-alpha” pathway suggests that these DEGs may modulate the innate immune response to viral infection, as interferon-alpha is a key cytokine that mediates antiviral activity and inflammation. Interferon-α production by plasmacytoid dendritic cells has been linked to acute kidney injury (Deng et al., 2021). Moreover, the weaker interferon antagonism of Omicron variant viruses compared to Delta variants suggests increased sensitivity to interferon treatment (Bojkova et al., 2022).

The “azurophil granule membrane” pathway implies that these DEGs may influence the function and activation of neutrophils, which are the most abundant type of white blood cells and play a crucial role in the defense against infection and tissue injury. Neutrophils, with their azurophils containing antimicrobial defensins, play a crucial role in both AKI and Omicron infection pathogenesis (Faurschou et al., 2002; Bolisetty and Agarwal, 2009; McKenna et al., 2022).

The “phospholipid scramblase activity” pathway suggests that these DEGs may regulate the exposure of phosphatidylserine on the cell surface, which is a marker of apoptosis and a trigger for phagocytosis and coagulation (Corneillie et al., 2023). These pathways may reflect the common mechanisms of cell death, inflammation, and vascular dysfunction that underlie AKI and Omicron infections. However, these findings are preliminary and require further research. The direct association of these factors and the potential therapeutic approaches need to be validated.

The principal KEGG pathway terms associated with the common DEGs encompassed Viral life cycle-HIV-1, Pertussis, Necroptosis, Influenza A, Complement and coagulation cascades, and COVID-19. These pathways may reflect the possible interactions and mechanisms of these DEGs in the pathogenesis and progression of AKI and Omicron infections. Previous studies have shown strong links between Pertussis, Necroptosis, and Influenza A with acute respiratory tract infections and severe inflammatory damage (Ismail et al., 2020; Fang et al., 2022; Kang and Wang, 2022; Matczak et al., 2022), which are also observed in COVID-19 and AKI. Necroptosis and COVID-19 are closely related, as the innate immune recognition of SARS-CoV-2 by multiple host cell pattern recognition receptors induces the release of pro-inflammatory cytokines and inflammatory cell death, leading to acute respiratory distress syndrome, tissue injury, and cytokine storms (Schifanella et al., 2023; Wiscovitch-Russo et al., 2023). The COVID-19 pandemic has significantly affected the spread of pertussis and the vaccination rates for pertussis-containing vaccines (Sandoval et al., 2023). However, there is a risk of misdiagnosing pertussis infections due to the overlap of cough symptoms between COVID-19 and pertussis.

In our endeavor to uncover common functional characteristics of proteins and identify potential therapeutic targets, we constructed a PPI network based on the DEGs. This methodology facilitated the identification of key hub genes, namely EIF2AK2, PLSCR1, GBP1, TNFSF10, C1QB, and BST2, which appear to bridge both conditions, suggesting their utility as potential therapeutic targets or biomarkers.

Notably, EIF2AK2 (Protein Kinase R), PLSCR1 (Phospholipid Scramblase 1), BST2 (Tetherin), and C1QB exhibited pronounced alterations in models of murine AKI, with strong correlations to the AKI marker Kim-1. This observation underscores the potential of these genes in influencing AKI’s progression. EIF2AK2, for instance, regulates the immune response and viral defenses through its activation by double-stranded RNA, leading to the phosphorylation of key proteins involved in initiating protein synthesis (Ge et al., 2021; Wei et al., 2023). Similarly, PLSCR1’s involvement in apoptosis and its interaction with viral proteins (Dal Col et al., 2022) suggest its role in controlling viral uptake and spread, evidenced by its identification as a restriction factor against SARS-CoV-2 (Xu et al., 2023), including the Omicron variant.

GBP1, through its GTPase activity, regulates cell death and has shown antiviral effects by promoting autophagy against pathogens (Zhang et al., 2021; Zhu et al., 2024). TNFSF10’s (TRAIL) recognized role in apoptosis further highlights the importance of cell death regulation in the context of AKI and viral infections (Dickinson et al., 2023), though its specific contributions require additional exploration. C1QB, integral to the complement system (Ji and Guo, 2023), implicates inflammation regulation as a crucial process in both AKI and viral infections. The ability of the Omicron variant to counteract BST2 emphasizes the latter’s role in viral infectivity and control (Zhao et al., 2022; Kitao et al., 2023).

This intricate interplay of immune response, apoptosis, and inflammation mediated by these hub genes provides essential insights into the pathogenesis of both AKI and Omicron infection. However, the detailed roles and mechanisms of these genes specifically in the context of AKI and Omicron infection remain to be elucidated.

We have also elucidated the intricate associations between Omicron infection and AKI through the interplay of TFs and miRNAs. TFs govern the expression of mRNA, while miRNAs regulate gene expression by means of RNA silencing at the post-transcriptional level (Politano et al., 2019; Liu et al., 2022). Consequently, TFs and miRNAs emerge as indispensable players in the development of diseases. Our comprehensive analysis has unraveled the intricate relationships among the commonly expressed differentially regulated genes (DEGs), TFs, and miRNAs. Among these, we have identified a cohort of TFs, namely GATA2, GATA3, FOXC1, TP53, STAT3, STAT1, CEBPB, ELK4, and REL, that demonstrate associations with diverse respiratory and kidney ailments such as COVID-19, asthma, and chronic kidney injury (Yu et al., 2017; Estrela et al., 2021; Fang et al., 2022; Yang et al., 2023; Zhang et al., 2023). In the context of establishing the relationship between DEGs and miRNAs, we have identified hsa-miR-129-2-3p, hsa-miR-130a-3p, hsa-miR-133a-3p, hsa-miR-146a-5p, and hsa-miR-124-3p as being significantly associated with the pathogenesis and exacerbation of Omicron infections and AKI. Previous investigations have linked these specific miRNAs to the progression and development of various inflammation-associated diseases, including asthma, chronic obstructive pulmonary disease, and colitis (Lindner et al., 2018; Zhai et al., 2020; Alamro et al., 2022; Qian et al., 2023a; Chen A. et al., 2023), thereby underscoring their potential roles in the context of Omicron infection and AKI. While our analysis sheds light on the potential involvement of these TFs and miRNAs, further experimental studies are imperative to validate their functional significance in the realm of Omicron infection and AKI.

Furthermore, our study has revealed intriguing connections between various diseases, such as Autosomal Recessive Predisposition, Myocardial Ischemia, Schizophrenia, Liver Cirrhosis, IgA Glomerulonephritis, Prostatic Neoplasms, and Influenza, and the hub genes identified. These findings suggest potential genetic associations with both AKI and Omicron infections. The diverse nature of these conditions points to a complex genetic landscape, where shared genetic elements may contribute to the susceptibility or severity of AKI and Omicron infections. However, the precise mechanistic links underlying these associations remain elusive. In the case of IgA Glomerulonephritis, AKI can occur when IgA deposits lead to a loss of kidney filtering ability (Wei et al., 2021; Li et al., 2023). Additionally, AKI exacerbations are often triggered by viral infections, including influenza (Mohammad et al., 2021; Birkelo et al., 2022). The co-infection risk between influenza A virus and SARS-CoV-2 is a significant concern for public health officials and clinicians (Solomon et al., 2020; Mehta et al., 2021; Qian et al., 2023b; Sun et al., 2023). Nonetheless, the absence of direct references linking these diseases to AKI and Omicron infections highlights the pioneering nature of our observations and the need for further exploration in this field.

In our quest to discover potential therapeutics for COVID-19 and AKI, we utilized network pharmacology analysis and pinpointed several promising candidates. Notably, Suloctidil, a well-established pharmaceutical, surfaced as a potential therapeutic agent for COVID-19 (Alegria-Arcos et al., 2022; Fang et al., 2022). Simultaneously, the FDA-approved HIV/AIDS medication, 3′-Azido-3′-deoxythymidine (AZT), has shown encouraging inhibitory effects on the RNA-dependent RNA polymerase (RdRp) of SARS-CoV, a critical component in the virus’s life cycle (Alegria-Arcos et al., 2022). Given the significant amino acid similarity (98%) between the RdRps of SARS-CoV and SARS-CoV-2 (So et al., 2021; Elfiky, 2022), it is reasonable to expect that AZT might exhibit a similar inhibitory effect on the polymerase of SARS-CoV-2.

Furthermore, our analysis unveiled several compounds that could potentially be therapeutically relevant for AKI and Omicron infection. These include Apocarotenal, beta-carotene, Selenium, Vigabatrin, Prenylamine, Isoetarine, and Terfenadine. Importantly, beta-carotene has been associated with renal protective mechanisms, possibly mediated by its antioxidant properties (Hosseini et al., 2010; Kaliappan et al., 2013). This discovery lays a promising foundation for its potential therapeutic application. However, it is crucial to conduct further in-depth investigations to clarify its exact role in the context of AKI and Omicron infection.

Given the complexity of AKI pathogenesis and the rapidly evolving nature of Omicron infection, a comprehensive understanding of the identified compounds is crucial. We suggest further scrutiny of each compound’s mode of action, potential adverse effects, and existing evidence of efficacy against AKI or Omicron infection. It is also essential to consider the potential synergy or antagonism between these compounds and other treatment modalities, framing them within the context of a comprehensive treatment strategy. This multifaceted approach will ensure we are well-prepared to address the challenges posed by AKI and the Omicron variant.

While this study offers initial insights, it is constrained by several critical limitations. Our bioinformatics findings necessitate thorough validation through conditional knockout mouse models and pharmacological assays to establish causality and evaluate clinical applicability. The proposed therapeutic targets demand comprehensive experimental assessment to ascertain their safety and efficacy prior to being considered for clinical application.

Furthermore, our research employs a mouse model of IR induced AKI to validate clinical data on COVID-19-associated AKI, acknowledging substantial differences in their pathogenesis and physiological responses. COVID-19-associated AKI is predominantly attributed to the virus’s direct cytopathic effects, systemic inflammation, and the ramifications of severe illness, such as hypoxia and shock (Matsumoto and Prowle, 2022). Conversely, the IR-induced AKI model in mice is defined by an abrupt, transient disruption in renal blood flow, followed by reperfusion and reoxygenation, triggering inflammatory and cellular responses (Chen J. et al., 2023). These variances underscore the need for caution in directly extrapolating our findings to the clinical management of COVID-19-associated AKI. Thus, the mouse model is delineated as an exploratory tool for uncovering potential molecular mechanisms and therapeutic avenues.

In essence, our findings should be regarded as preliminary indicators that illuminate avenues for subsequent research rather than conclusive evidence. This investigation highlights potential pathways and biomarkers, yet it necessitates rigorous *in vitro* and *in vivo* experimentation to translate these initial observations into practical diagnostic and therapeutic strategies for acute kidney injury in the context of the COVID-19 pandemic. In addition to the aforementioned findings, our validation dataset shows that the expression levels of the four hub genes (PLSCR1, GBP1, BST2, and C1QB) are consistent with our hub results, while differences are observed in the other two hub genes (EIF2AK2 and TNFSF10). This difference can be attributed to the heterogeneity of patient samples and the complex regulatory network involved in gene regulation. To further deepen our research, we plan to collect clinical data related to patients with AKI and correlate these data with changes in gene expression. This will become a new focus of our ongoing research.

## 5 Conclusion

In conclusion, this study provides preliminary insights into the possible molecular links between AKI and Omicron infection, both primary and secondary, by revealing common pathways and markers. We identified shared DEGs between Omicron infection and AKI, and performed gene expression analysis to discover relevant GO terms and intracellular signaling pathways. We also constructed a PPI network using the common DEGs, and determined the top six hub genes. Furthermore, we discovered various drugs and their interactions with the hub genes, which could serve as potential therapeutic targets. While many vaccines are currently used to fight Omicron infection, the constant mutation of SARS-CoV-2 demands continuous efforts to develop effective vaccines and treatments for COVID-19. Considering that AKI patients may be more susceptible to Omicron infection, especially after repeated exposure, our findings, including hub genes, potential drugs, and possible regulatory TFs and miRNAs, offer critical knowledge for developing new drugs or repurposing existing ones to address concurrent AKI and Omicron infection. However, these findings are initial and require further research. The targeting of the identified biomarkers and the utilization of the suggested drugs must be approached with caution, as more exploration and validation are needed to confirm their safety and efficacy. This study contributes to our evolving understanding of the complex interplay between the molecular mechanisms and the pathogenesis of AKI and Omicron infection. It is a stepping stone rather than a definitive solution, and it highlights the need for continued, comprehensive research to pave the way for more effective and validated treatment strategies in the future.

## Data Availability

The datasets presented in this study can be found in online repositories. The names of the repository/repositories and accession number(s) can be found in the article/Supplementary Material.
