# Supplementary material for: Deciphering the molecular nexus between Omicron infection and acute kidney injury: a bioinformatics approach
Source: Front Mol Biosci. 2024 Jul 4;11:1340611. doi: 10.3389/fmolb.2024.1340611 (PMC11254815; doi:10.3389/fmolb.2024.1340611)
Supplement: Supplementary file 1 [file Table1.DOCX]

**Supplementary materials**

**Table S1** | Common genes of AKI and Omicron infections (*n* = 9).

| Symbol | Gene ID | Gene name |
| --- | --- | --- |
| BST2 | 684 | Bone marrow stromal cell antigen 2 |
| C1QB | 713 | Complement C1q B chain |
| EIF2AK2 | 5610 | Eukaryotic translation initiation factor 2 alpha kinase 2 |
| GBP1 | 2633 | Guanylate binding protein 1 |
| MYOF | 26509 | Myoferlin |
| PHF24 | 23349 | PHD finger protein 24 |
| PLSCR1 | 5359 | Phospholipid scramblase 1 |
| TMEM123 | 114908 | Transmembrane protein 123 |
| TNFSF10 | 8743 | TNF superfamily member 10 |

**Table S2** | TF-Gene topology table.

| Id | Label | Degree | Betweenness |
| --- | --- | --- | --- |
| 5610 | EIF2AK2 | 10 | 210.17 |
| 8743 | TNFSF10 | 8 | 192.03 |
| 2633 | GBP1 | 7 | 150.5 |
| 713 | C1QB | 6 | 133.7 |
| 5359 | PLSCR1 | 5 | 105.6 |
| 2624 | GATA2 | 4 | 203.4 |
| 2300 | FOXL1 | 3 | 122.8 |
| 2296 | FOXC1 | 2 | 37.1 |
| 5452 | POU2F2 | 2 | 37.1 |
| 2005 | ELK4 | 2 | 23.6 |
| 5966 | REL | 1 | 0 |
| 6689 | SPIB | 1 | 0 |
| 1482 | NKX2-5 | 1 | 0 |
| 1051 | CEBPB | 1 | 0 |
| 3725 | JUN | 1 | 0 |
| 1385 | CREB1 | 1 | 0 |
| 7528 | YY1 | 1 | 0 |
| 4205 | MEF2A | 1 | 0 |
| 3202 | HOXA5 | 1 | 0 |
| 5468 | PPARG | 1 | 0 |
| 2625 | GATA3 | 1 | 0 |
| 1869 | E2F1 | 1 | 0 |
| 25988 | HINFP | 1 | 0 |
| 7157 | TP53 | 1 | 0 |
| 7392 | USF2 | 1 | 0 |
| 6772 | STAT1 | 1 | 0 |
| 7003 | TEAD1 | 1 | 0 |
| 2002 | ELK1 | 1 | 0 |
| 2099 | ESR1 | 1 | 0 |
| 8626 | TP63 | 1 | 0 |
| 6721 | SREBF2 | 1 | 0 |
| 2908 | NR3C1 | 1 | 0 |
| 2019 | EN1 | 1 | 0 |

**Table S3** | MiRNA-Gene topology table.

| Id | Label | Degree | Betweenness |
| --- | --- | --- | --- |
| 5610 | EIF2AK2 | 53 | 3505.5 |
| 684 | BST2 | 41 | 2490.01 |
| 8743 | TNFSF10 | 26 | 1712.13 |
| 2633 | GBP1 | 23 | 1059.83 |
| 5359 | PLSCR1 | 22 | 1012.08 |
| MIMAT0000422 | hsa-mir-124-3p | 6 | 436.96 |
| MIMAT0004605 | hsa-mir-129-2-3p | 6 | 436.96 |
| MIMAT0000425 | hsa-mir-130a-3p | 5 | 389.99 |
| MIMAT0000427 | hsa-mir-133a-3p | 5 | 389.99 |
| MIMAT0000449 | hsa-mir-146a-5p | 4 | 227.87 |
| MIMAT0000264 | hsa-mir-203a-3p | 4 | 176.14 |
| MIMAT0004494 | hsa-mir-21-3p | 4 | 176.14 |
| MIMAT0004501 | hsa-mir-27a-5p | 4 | 176.14 |
| 713 | C1QB | 4 | 25.45 |
| MIMAT0000082 | hsa-mir-26a-5p | 3 | 125.89 |
| MIMAT0000681 | hsa-mir-29c-3p | 3 | 124.42 |
| MIMAT0000069 | hsa-mir-16-5p | 3 | 112.78 |
| MIMAT0000646 | hsa-mir-155-5p | 3 | 108.2 |
| MIMAT0001541 | hsa-mir-449a | 3 | 108.2 |
| MIMAT0003327 | hsa-mir-449b-5p | 3 | 108.2 |
| MIMAT0000416 | hsa-mir-1-3p | 3 | 78.11 |
| MIMAT0000269 | hsa-mir-212-3p | 3 | 78.11 |
| MIMAT0000458 | hsa-mir-190a-5p | 2 | 69.43 |
| MIMAT0004929 | hsa-mir-190b | 2 | 69.43 |
| MIMAT0000088 | hsa-mir-30a-3p | 2 | 69.43 |
| MIMAT0000693 | hsa-mir-30e-3p | 2 | 69.43 |
| MIMAT0000259 | hsa-mir-182-5p | 2 | 57.61 |
| MIMAT0000727 | hsa-mir-374a-5p | 2 | 57.61 |
| MIMAT0001341 | hsa-mir-424-5p | 2 | 57.61 |
| MIMAT0000252 | hsa-mir-7-5p | 2 | 34.89 |
| MIMAT0019776 | hsa-mir-1343-3p | 2 | 20.28 |
| MIMAT0000254 | hsa-mir-10b-5p | 2 | 20.28 |
| MIMAT0000724 | hsa-mir-372-3p | 2 | 20.28 |
| MIMAT0000267 | hsa-mir-210-3p | 2 | 20.15 |
| MIMAT0000083 | hsa-mir-26b-5p | 2 | 4.48 |
| MIMAT0000096 | hsa-mir-98-5p | 1 | 0 |
| MIMAT0000417 | hsa-mir-15b-5p | 1 | 0 |
| MIMAT0000426 | hsa-mir-132-3p | 1 | 0 |
| MIMAT0000728 | hsa-mir-375 | 1 | 0 |
| MIMAT0000731 | hsa-mir-378a-5p | 1 | 0 |
| MIMAT0022492 | hsa-mir-5699-3p | 1 | 0 |
| MIMAT0000432 | hsa-mir-141-3p | 1 | 0 |
| MIMAT0000434 | hsa-mir-142-3p | 1 | 0 |
| MIMAT0000435 | hsa-mir-143-3p | 1 | 0 |
| MIMAT0004658 | hsa-mir-155-3p | 1 | 0 |
| MIMAT0000068 | hsa-mir-15a-5p | 1 | 0 |
| MIMAT0000071 | hsa-mir-17-3p | 1 | 0 |
| MIMAT0000261 | hsa-mir-183-5p | 1 | 0 |
| MIMAT0000456 | hsa-mir-186-5p | 1 | 0 |
| MIMAT0000072 | hsa-mir-18a-5p | 1 | 0 |
| MIMAT0000461 | hsa-mir-195-5p | 1 | 0 |
| MIMAT0000682 | hsa-mir-200a-3p | 1 | 0 |
| MIMAT0000265 | hsa-mir-204-5p | 1 | 0 |
| MIMAT0004500 | hsa-mir-26b-3p | 1 | 0 |
| MIMAT0000688 | hsa-mir-301a-3p | 1 | 0 |
| MIMAT0000710 | hsa-mir-365a-3p | 1 | 0 |
| MIMAT0022834 | hsa-mir-365b-3p | 1 | 0 |
| MIMAT0004956 | hsa-mir-374b-3p | 1 | 0 |
| MIMAT0004955 | hsa-mir-374b-5p | 1 | 0 |
| MIMAT0003150 | hsa-mir-455-5p | 1 | 0 |
| MIMAT0002820 | hsa-mir-497-5p | 1 | 0 |
| MIMAT0002874 | hsa-mir-503-5p | 1 | 0 |
| MIMAT0004801 | hsa-mir-590-3p | 1 | 0 |
| MIMAT0004810 | hsa-mir-629-5p | 1 | 0 |
| MIMAT0005949 | hsa-mir-664a-3p | 1 | 0 |
| MIMAT0004982 | hsa-mir-939-5p | 1 | 0 |
| MIMAT0004985 | hsa-mir-942-5p | 1 | 0 |
| MIMAT0004987 | hsa-mir-944 | 1 | 0 |
| MIMAT0000444 | hsa-mir-126-5p | 1 | 0 |
| MIMAT0000430 | hsa-mir-138-5p | 1 | 0 |
| MIMAT0005451 | hsa-mir-522-5p | 1 | 0 |
| MIMAT0022723 | hsa-mir-548h-3p | 1 | 0 |
| MIMAT0018446 | hsa-mir-548z | 1 | 0 |
| MIMAT0000424 | hsa-mir-128-3p | 1 | 0 |
| MIMAT0000460 | hsa-mir-194-5p | 1 | 0 |
| MIMAT0000084 | hsa-mir-27a-3p | 1 | 0 |
| MIMAT0000245 | hsa-mir-30d-5p | 1 | 0 |
| MIMAT0000062 | hsa-let-7a-5p | 1 | 0 |
| MIMAT0000063 | hsa-let-7b-5p | 1 | 0 |
| MIMAT0000064 | hsa-let-7c-5p | 1 | 0 |
| MIMAT0000066 | hsa-let-7e-5p | 1 | 0 |
| MIMAT0000067 | hsa-let-7f-5p | 1 | 0 |
| MIMAT0000414 | hsa-let-7g-5p | 1 | 0 |
| MIMAT0000415 | hsa-let-7i-5p | 1 | 0 |
| MIMAT0004551 | hsa-mir-30d-3p | 1 | 0 |
| MIMAT0005889 | hsa-mir-548l | 1 | 0 |
| MIMAT0000070 | hsa-mir-17-5p | 1 | 0 |
| MIMAT0000617 | hsa-mir-200c-3p | 1 | 0 |
| MIMAT0000075 | hsa-mir-20a-5p | 1 | 0 |
| MIMAT0002852 | hsa-mir-517a-3p | 1 | 0 |
| MIMAT0003880 | hsa-mir-671-5p | 1 | 0 |
| MIMAT0005924 | hsa-mir-1270 | 1 | 0 |
| MIMAT0000451 | hsa-mir-150-5p | 1 | 0 |
| MIMAT0000273 | hsa-mir-216a-5p | 1 | 0 |
| MIMAT0000085 | hsa-mir-28-5p | 1 | 0 |
| MIMAT0000086 | hsa-mir-29a-3p | 1 | 0 |
| MIMAT0000100 | hsa-mir-29b-3p | 1 | 0 |
| MIMAT0000764 | hsa-mir-339-5p | 1 | 0 |
| MIMAT0003238 | hsa-mir-573 | 1 | 0 |
| MIMAT0003887 | hsa-mir-769-3p | 1 | 0 |
| MIMAT0002176 | hsa-mir-485-3p | 1 | 0 |
| MIMAT0004926 | hsa-mir-708-5p | 1 | 0 |
| MIMAT0000418 | hsa-mir-23b-3p | 1 | 0 |
| MIMAT0000440 | hsa-mir-191-5p | 1 | 0 |
| MIMAT0000462 | hsa-mir-206 | 1 | 0 |
| MIMAT0000271 | hsa-mir-214-3p | 1 | 0 |
| MIMAT0000278 | hsa-mir-221-3p | 1 | 0 |
| MIMAT0000255 | hsa-mir-34a-5p | 1 | 0 |
| MIMAT0000685 | hsa-mir-34b-5p | 1 | 0 |
| MIMAT0000686 | hsa-mir-34c-5p | 1 | 0 |
| MIMAT0000726 | hsa-mir-373-3p | 1 | 0 |
| MIMAT0005891 | hsa-mir-1303 | 1 | 0 |
| MIMAT0004503 | hsa-mir-29a-5p | 1 | 0 |
| MIMAT0018104 | hsa-mir-3679-5p | 1 | 0 |
| MIMAT0001635 | hsa-mir-452-5p | 1 | 0 |
| MIMAT0002846 | hsa-mir-520c-3p | 1 | 0 |
